# In Poor Health

**Published:** 1876-06

**Authors:** 


### *IN POOR HEALTH.*

How often do we hear this reply in answer to inquiries after the health of individuals. We hear it so constantly that we sometimes think that invalidity is the normal condition of the human race. Health is only truly prized when it is gone. If the system is in perfect order, we are almost unconscious of the existence of the vital organs. The machinery of life moves noiselessly and harmoniously, and the mind is in sympathy with it; we are hopeful, cheerful, and happy. But let there be any derangement in the machinery, and all is changed. Mind and body both suffer to an extent depending upon the gravity of the physical derangement, from a feeling of sadness to that which contemplates self-destruction.

This state of things will exist until the masses become better informed in regard to the laws of health and life; until they understand that every violation of these laws demands a penalty, which must be paid with interest; too often after the prime of life, when its vigor and powers are so feeble as to be unable to offer much resistance to the march of disease, and the victim sinks helplessly into his grave, at an age when, with proper care he might have been tranquilly enjoying the harvest of a prudent life. More than half of the diseases that end fatally before middle age may be traced directly back to abuses of the stomach. That unfortunate organ is very generally made the receptacle for all sorts of crude and indigestible things, forced into it in all sizes and shapes that the narrow passage of the throat will admit, and at all hours. No machinery made by human hands could so long endure this treatment. But the stomach faithfully endeavors to do its work. It labors vigorously to digest all that is forced into it; but finally, when taxed beyond endurance, it is compelled to exhibit weakness. Then the liver becomes torpid; the bowels cease to act regularly and healthfully; effete matters are absorbed into the system, poisoning the blood, and bringing on a train of evils that will sooner or later make wreck of constitutions otherwise the soundest and best. The remedy for these evils should be applied in infancy. Parents should see that their children are provided with wholesome food, the supply of which should not be stinted—no child was ever injured by having all the really wholesome food it craved. We don't believe in short rations, for old or young. But above all, parents should see that their children form the habit of going to the closet at least once a day, and at a regular hour. Such a habit once established, will continue through life, and will save years of misery and premature death. The demands of our physical well being are neither onerous or unreasonable. That they should be so generally disregarded, when so much in life that is worth living for depends upon them, is one of the mysteries we could never understand.

